# A PUF- and Biometric-Based Lightweight Hardware Solution to Increase Security at Sensor Nodes

**DOI:** 10.3390/s18082429

**Published:** 2018-07-26

**Authors:** Rosario Arjona, Miguel Ángel Prada-Delgado, Javier Arcenegui, Iluminada Baturone

**Affiliations:** Instituto de Microelectrónica de Sevilla (IMSE-CNM), Consejo Superior de Investigaciones Científicas (CSIC), Universidad de Sevilla, Américo Vespucio, 28, 41092 Seville, Spain; prada@imse-cnm.csic.es (M.A.P.-D.); arcenegui@imse-cnm.csic.es (J.A.); lumi@imse-cnm.csic.es (I.B.)

**Keywords:** security for sensor networks, trusted sensor nodes, Physically Unclonable Functions (PUFs), SRAM PUFs, lightweight biometrics, fingerprint recognition, multibiometrics, low-power microcontrollers

## Abstract

Security is essential in sensor nodes which acquire and transmit sensitive data. However, the constraints of processing, memory and power consumption are very high in these nodes. Cryptographic algorithms based on symmetric key are very suitable for them. The drawback is that secure storage of secret keys is required. In this work, a low-cost solution is presented to obfuscate secret keys with Physically Unclonable Functions (PUFs), which exploit the hardware identity of the node. In addition, a lightweight fingerprint recognition solution is proposed, which can be implemented in low-cost sensor nodes. Since biometric data of individuals are sensitive, they are also obfuscated with PUFs. Both solutions allow authenticating the origin of the sensed data with a proposed dual-factor authentication protocol. One factor is the unique physical identity of the trusted sensor node that measures them. The other factor is the physical presence of the legitimate individual in charge of authorizing their transmission. Experimental results are included to prove how the proposed PUF-based solution can be implemented with the SRAMs of commercial Bluetooth Low Energy (BLE) chips which belong to the communication module of the sensor node. Implementation results show how the proposed fingerprint recognition based on the novel texture-based feature named QFingerMap16 (QFM) can be implemented fully inside a low-cost sensor node. Robustness, security and privacy issues at the proposed sensor nodes are discussed and analyzed with experimental results from PUFs and fingerprints taken from public and standard databases.

## 1. Introduction

Security is becoming greatly important in sensor networks since they are employed in critical tasks such as military, industrial, home, and health applications, among others. Security goals usually addressed are confidentiality, integrity, freshness, authentication, and non-repudiation. The data sensed and transmitted should be confidential if they are related to sensitive information (as is the case with geolocation data in the deployment of military forces) and should maintain integrity to prove they have not been altered or introduced by an adversary. Data confidentiality and integrity can be achieved by symmetric key cryptography, which uses the same key for encryption and decryption. Symmetric key cryptography is preferred to asymmetric or public key cryptography at sensor nodes because it requires fewer computation resources and lower power consumption. As drawback, key management is more complex in symmetric key cryptography. A widely adopted solution, suitable for sensor nodes, is a key pre-distribution scheme that stores a master secret key in all sensor nodes before they are deployed. After deployed, any pair of sensor nodes can use the global master secret key to negotiate a new shared key [1]. The problem of using a master key is that the whole network security can be compromised if a sensor node is compromised. Hence, highly secure non-volatile memories can be employed to store the secret key. However, they are more expensive than standard memories and several techniques have been reported to attack and extract data from them [2]. Trusted hardware can be used for secure storage [3,4,5]. However, it is impractical for low-cost sensor nodes. Furthermore, malware has been demonstrated to be run in these hardware enclaves [6].

Instead of storing a secret key, the solution proposed in this paper is to reconstruct it by using a bit string obtained from the physical identity of the sensor node. A recent research area is to obtain bit strings from Physically Unclonable Functions (PUFs), which allow generating unique, distinctive and unpredictable identifiers. They are produced by the manufacturing process variability of the sensor hardware [7]. A sensor with PUFs cannot be cloned physically because the manufacturing process introduces variations that are specific to each sensor. Hence, a compromised-key attack is more difficult (because the key is not stored anyway). In addition, counterfeiting (false copies of sensors) attacks are more difficult because the physical identity of the sensor node can be authenticated [8].

Among the electronic circuits employed as PUFs (SRAMs, latches, D Flip-Flops, arbiters, ring oscillators, etc.), SRAM PUFs are used in this work since no additional circuitry is needed. Commonly, SRAMs are readily present in sensors. Hence, using SRAM PUFs is low-cost from the hardware point of view. The use of SRAMs as PUFs involves reading the start-up values when the memory is switched on and no data is written. The start-up values of one SRAM are difficult to be generated by another SRAM or ascertained by someone willing to clone the SRAM [9].

A well-known approach to reconstruct keys from noisy data (such as those provided by PUFs) is the use of Helper Data algorithms [10,11]. They are based on the storage of ideally non-sensitive information named as Helper Data. Sensitive information such as the secret key is obfuscated with the PUF response to generate Helper Data that do not reveal information about the secret key. Helper Data are generated and stored at an enrollment stage. Then, whenever the key is required, a key reconstruction stage is carried out. The most popular scheme is the Code Offset-based Helper Data algorithm [11], where an Error Correcting Code (ECC) is employed to cope with noise. The secret key is encoded according to the ECC. At key reconstruction, a new PUF response is obtained that will not be exactly the same as the response used at the enrolment stage but very similar. Using the new response and the stored Helper Data, a noisy version of the encoded secret key is obtained. Then, the decoder of the ECC is able to recover the secret key. This paper proposes a low-cost solution to carry out these stages at sensor nodes.

Many applications of sensor networks not only require trusted sensors but also that genuine individuals prove with their physical presence that they are associated with the sensed data. For example, a soldier wearing the sensor node can prove that the geolocation data transmitted are associated with him/her and not with an enemy that could have stolen it [12]. These sensor nodes can be considered as nodes with dual-factor authentication. Individual recognition at sensor nodes allows high-level security from the origin of the data, which is interesting to create a digital chain of custody [13]. Neither a genuine sensor employed by a non-legitimate individual nor a fake sensor employed by a legitimate individual are accepted. If the dual-authentication is carried out at sensor node, the security perimeter is constrained to the node. Hence, the number of possible attacks is necessarily smaller.

As PUFs authenticate the physical identity of the sensor, biometrics authenticates the physical presence of the user through physical, physiological or behavioral traits [14]. Examples of biometric traits are fingerprints or faces (as physical traits); heart or brain signals (as physiological traits); and gait or handwritten signatures (as behavioral traits). Among the biometric features available, fingerprints are employed in this work because they are general (every individual possesses fingers except in rare cases), very distinctive (even for twins), and permanent along the life of an individual [15]. Besides, small and low-cost fingerprint sensors can capture high-quality fingerprint images. Taking into account that sensor nodes have limited computational, memory, and power resources, this paper proposes a lightweight fingerprint recognition solution which considers a novel texture-based fingerprint feature named QFingerMap16, herein referred to as QFM. It is obtained after applying very simple arithmetic and logic operations at all the processing steps so that it can be implemented in low-cost sensor nodes. The proposal is able to provide good accuracy in recognition by using several fingers and samples per finger. In addition, the solution allows user interaction and collaboration by informing about the quality and evolution of the feature extraction process.

If the network can verify both the identity of the sensor that transmits the data and the individual that uses it, repudiation attacks are more difficult because a node and a user cannot deny having sent a message already sent. Other entities can prove that sensor with the presence of that user have sent the measurement. Impersonation attacks (in which a fake sensor node assumes the identity of one of the legitimate sensors in the network or an impostor user assumes the identity of one of the legitimate users) are more difficult.

Like the secret key used in the cryptographic algorithms, the biometric templates of the individuals are sensitive information that should not be stored in the sensor [16,17,18]. Biometric salting techniques add random information (salt) to increase the entropy of the biometric template and generate a transformed template. A well-known technique is BioHashing, which generates a transformed template from the biometric template and random data contained in a token, smart card, etc. [18]. Instead of using random data stored in the sensor node and complex transformations of the biometric template, the solution proposed in this paper is to use again the PUF responses of the sensor, which is more secure because they are not stored anywhere. Besides, the QFM-based features are employed without any transformation, which allows a lightweight hardware solution. At the biometric enrolment stage (which should be carried out in a secure way whenever an individual is associated with a sensor), non-sensitive Salted Data are generated and stored in the sensor. Ideally, these biometric Salted Data do not leak information about the biometric template as the Helper Data associated with the secret key do not leak information about the secret key. At the biometric verification stage (which is carried out whenever an individual should be recognized at the sensor node), the biometric Salted Data stored are employed to compare the template biometric features with the query biometric features obtained from the individual.

In summary, the contributions of this paper are the following:

1. The proposal of sensor nodes authenticated by their physical identity and the physical presence of their users.
2. A low-cost solution that uses the SRAM PUF of the sensor node to prove the physical identity of the node, to obfuscate secret keys, and to obtain the nonces of the proposed communication protocol.
3. The proposal of a lightweight fingerprint recognition solution that proves the physical presence of the user and that protects biometric data with the PUF response at the sensor node.
4. Experimental results showing that the proposed solutions can be implemented in low-cost hardware platforms.

To the best of our knowledge, this is the first work which combines PUFs and biometrics to increase security at sensor nodes. The paper is structured as follows: Section 2 summarizes several application scenarios of the proposed sensors. Section 3 presents a low-cost solution to use the SRAM of the sensor nodes to provide not only physical identities of devices but also obfuscation of sensitive information and nonces. Section 4 describes the fingerprint feature named QFingerMap16 (QFM), how it can be extracted using sensor nodes with low-cost hardware, and how it can be protected at sensor nodes so that biometric data are always protected whenever sent and stored. Section 5 describes a communication protocol between the proposed sensor nodes and the base station. Results in terms of authentication, implementation, security and privacy are presented and discussed in Section 6. Finally, conclusions are given in Section 7.

## 2. Application Scenarios

The proposed solution finds application in those scenarios where sensor nodes associated with individuals transmit measurements that require high security against counterfeiting attacks to sensor nodes and against repudiation and impersonation attacks to both sensors and individuals. Several examples are summarized in the following.

### 2.1. Sensor Nodes in the Industrial Internet of Things (IIoT)

An illustrative scenario is the IIoT. Cyber-attacks in the industrial domain have been mostly focused on the loss of data. However, since sensors allow IIoT devices to interact with the physical world, cyber-attacks can enter now into the physical world and, hence, can cause physical harm. A chemical or a nuclear plant or a multimillion dollar industrial process can be destroyed or lead to threaten the lives of their employees and citizens in the neighborhood.

Ciphering data (which reduces the possibility of transmitting false data) may not be sufficient in this scenario. Avoiding counterfeiting is very important because fake sensors do not meet usually the required quality provided by legitimate sensors. In addition, fake sensors can be the trapdoor for industrial espionage. If the sensor nodes proposed herein are copied, the resulting fake sensors cannot impersonate them because it is not possible to clone the manufacturing properties of the device to generate the same PUF. In this case, they are unable to recover the secret key from the Helper Data stolen.

Since many sensors involved with critical tasks should provide high precision and resolution, they should be calibrated and tested regularly on-site by accredited professionals. During calibration and testing, it is very important to avoid impersonation attacks of adversaries assuming the identity of accredited professionals. Otherwise, adversaries or competitors would be able to modify maliciously the sensor behavior.

Avoiding repudiation attacks is also very important, because an accredited professional may become malicious. If the proposed sensors are used by this attacker, the base station can prove that the transmitted data came from that sensor used by that individual.

### 2.2. Sensor Nodes Used for Regulation Compliance

Many regulations that prevent occupational and environmental risks establish that the measurements of certain variables should meet accepted levels (for example, levels of noise, radioactivity, carbon-dioxide emissions, etc.). Environmental, occupational, health and safety inspectors perform on-site analysis of those variables to prevent harm to the environment, citizens, and workers. They wear sensor nodes conveniently calibrated and tested to take the needed measurements.

Since the sensors proposed herein send ciphered measurements to a base station, falsification of the data in order to bypass the regulations is more difficult. In this scenario, ciphering may not be enough. Fake sensors measuring false data as well as impostor inspectors assuming the identity of a legitimate one should be avoided because otherwise, dangerous environments can be certified as healthy. Repudiation attacks are reduced because it can also be proven that wrong or malicious messages were sent by the sensor of an inspector who carried out wrong evaluations or who became malicious.

### 2.3. Sensor Nodes into Soldier Uniforms

Integration of sensors into soldier uniforms reduces greatly the weight burden placed on them and provides more opportunities to successfully execute missions. Sensors can measure not only geolocation data of soldiers but also health and body conditions, such as heart rate, breathing or blood pressure. Rapid access to this information could help soldiers and officers in charge of missions survive otherwise lethal enemy attacks.

Since this information is sensitive, it should be transmitted ciphered. However, ciphering may not be sufficient. Fake sensors should not be used in this scenario.

Using the proposed sensors an enemy cannot impersonate a soldier because his/her biometric features do not match with the template of the legitimate soldier. The base station can track the messages sent by the soldiers wearing the sensors and detect if he/she is executing correctly the mission or there is something wrong (non-repudiation is achieved).

### 2.4. Sensor Nodes Used for Arms Control

The question of arms control is of great significance to peace, security and stability. Arms should be identified to prevent their negligent or illicit use, manufacturing, and trafficking. A sensor node embedded in an arm is very interesting to conduct ballistic tests (distance, direction, and behavior of their shots) or to detect if an arm goes outside a controlled area. People authorized to use arms are identified by their arm license.

If the sensor nodes proposed herein are embedded into arms they could identify both the arm and his/her user. Since the sensors prove their authenticity, they can prove the arm is legal. If they send ciphered measurements, false data sent by illegal arms can be detected more easily. The arm can be assigned to an individual (authorized by an arm license) who is registered at the sensor node to use the arm. If the arm is stolen or lost, there is no risk of being used by a non-authorized individual. Concerning forensic analysis, a digital chain of custody can be achieved if arms are equipped with the proposed sensors since the base station can prove which data are sent by the sensor embedded in the arm so that repudiation of criminal acts is more difficult.

## 3. Trusted Sensor Nodes Based on SRAM PUFs

### 3.1. Physical Identities of Sensor Nodes Based on SRAM PUFs

SRAMs are composed of memory cells arranged in large memory array structures. Each SRAM cell is a bistable circuit whose logic memory functionality comes from two cross-coupled inverters. A write operation forces the SRAM cell to transition towards one of the two stable states. If the cell is powered-up and no write operation is carried out, the positive feedback between the two inverters leads the cell to the start-up value imposed by the inverter which begins to conduct. The conditions that make one inverter be the winner can be intrinsic or external. Intrinsic conditions are related to mismatching between the inverters due to the variability of semiconductor fabrication process. External conditions are aging, power supply voltage value (Vdd), ambient temperature, and ramp-up time (i.e., the time to reach Vdd after power-on), being the two last ones the most influential [8,9]. The start-up values of SRAM cells have been used to identify the SRAM because the intrinsic often dominate over the external conditions in most cells. Hence, although the external conditions change, the SRAM intrinsic nature remains. If the intrinsic conditions are random, due to fabrication process variability, the start-up values of one SRAM are difficult to be generated by another SRAM or ascertained by someone willing to clone the SRAM. Hence, SRAMs behave as a PUF and can be identified by their start-up values (the PUF response). The start-up values of the memory cells of two different SRAMs are different (which is known as PUF uniqueness) and the start-up values in a specific SRAM are almost the same for each powered-up (which is known as PUF reliability).

A sensor node has typically several parts: a communication module, a main microcontroller, and an energy source (a battery or an embedded form of energy harvesting). SRAMs which can be powered down and up to act as PUFs can be found, for example, in the communication module. The sensor can power down this module at certain times when no communication is carried out to save power (since sensor nodes have limited power budget). When communication is needed, the module is powered up and this can be exploited to obtain the SRAM start-up values.

The difference between two PUF responses obtained from SRAM start-up values is measured by computing their Hamming distance (Hdist). In other words, Hdist computes the number of bits that are different in two PUF responses.

Experimentally, given *k* sequences, *R*, of *n* bits generated by the start-up values of SRAM cells, the average fractional Hdist is calculated as follows:

(1) $${\frac{Hdist}{n}]}_{avg}\;=\frac{2}{k\cdot \left(k-1\right)\cdot n}\cdot \sum_{i=1}^{k-1}\sum_{j=i+1}^{k}Hdist\left(R_{i},R_{j}\right).$$

A condition for ideal PUF uniqueness is that PUF responses from different memories (known as the impostor population of PUF responses, in recognition applications) are quite different and the average fractional Hdist is therefore 0.5.

Concerning PUF reliability, if a SRAM PUF is completely reliable, all the PUF responses from the same memory (known as the genuine population of PUF responses, in recognition applications) should be the same, and the average fractional Hdist will therefore be zero. However, Hdist between two genuine responses is usually small but not zero. The reason is that most of the SRAM cells have a strong tendency to a preferred start-up value (they will be named herein as STB cells or stable cells). However, there are also a few cells that have a weak tendency or no preference at all (which will be named herein as RND cells or random cells). They provide different start-up values (known as flipping bits), due to variations of external conditions. The average probability that a start-up value in the genuine PUF responses may change (the bit error or bit flipping probability *p*) can be estimated by the average fractional Hdist in (1) computed for the genuine population of PUF responses. The comparison between each pair of bits in the PUF responses is modeled essentially as a Bernoulli trial, which takes value ‘1′ (if the bit changes) with probability *p* and a value of ‘0′ (if the bit does not change) with probability *1 − p*. If the *n* bits obtained from the start-up values of *n* cells are assumed to be independent, the probability of finding *t* flipping bits (or errors) in them is given by a binomial distribution, as follows:

(2) $$P\left(t\right)=\left(\begin{array}{c} n\\ t \end{array}\right)\cdot p^{t}\cdot {\left(1-p\right)}^{n-t}.$$

Hence, the probability that a PUF response of *n* bits contains more than *t* flipping bits is given by:

(3) $$P_{total}=1-\sum_{i=0}^{t}\left(\begin{array}{c} n\\ i \end{array}\right)\cdot p^{i}\cdot {\left(1-p\right)}^{n-i}.$$

In order to cope with the noise of genuine PUF responses, Error Correcting Codes (ECCs) are employed. A model using Equations (1)–(3) is used by many authors to select the most suitable ECC according to the bit error probability, *p*, in the PUF response [9,11]. Given *p* (estimated by the average fractional Hdist in (1) for the genuine population) and given an ECC (with *n*-bit codewords and capacity to correct up to *t* errors), the capability of the ECC to achieve a given $P_{total}$ can be evaluated with Equation (3). For a given $P_{total}$, the number of errors (flipping bits) to be corrected and, hence, the complexity of the ECC, decreases as the value of the average fractional Hdist for the genuine population gets lower. This is why using only STB cells instead of all SRAM cells is better to obfuscate secrets, as proposed in the following section.

### 3.2. A Low-Cost Solution to Obfuscate Sensitive Information through SRAM PUFs

One of the applications of PUFs is low-cost obfuscation of secrets that can be recovered from new PUF responses. Helper-Data algorithms are employed for this purpose. They consist of two stages, Helper-Data generation and secret reconstruction. The solution proposed in this paper is that, previously, a first registration phase is carried out to store in the sensor the masks which classify the SRAM cells as ID cells (identifier cells) and RND cells (random cells). This first registration phase is performed only once. In the other side, Helper-Data generation is performed whenever it is required to change the Helper Data because another secret has to be obfuscated, and secret reconstruction is performed whenever the secret is needed.

**Figure 1:**
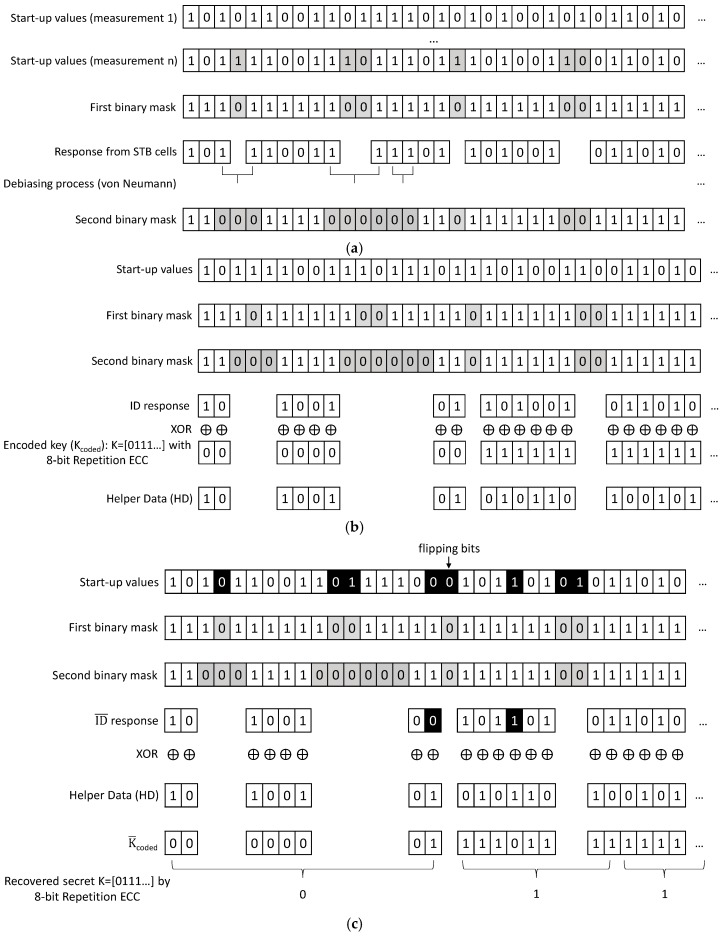
(**a**) Generation of masks for ID and RND cells in the first registration phase; (**b**) Helper-Data generation stage; (**c**) Secret reconstruction stage by using Helper Data and masks.

The first registration phase is carried out before sensor nodes are deployed, in a secure environment. The SRAM PUF of the sensor node is powered down and up several times and their start-up values are compared. The cells that do not change their start-up values are classified as STB cells (stable cells) [8,9]. A first binary mask identifies with a ‘1′ the STB cells. This is shown in the upper part of Figure 1a.

Then, the start-up values of the STB cells are debiased by the pair-output von Neumann or 2O-VN debiasing algorithm proposed in [19] to obtain a PUF response not only with low bit flipping but also without bias. The STB cells that provide a debiased response, which will be named herein as ID cells, are identified with a ‘1′ by a second binary mask. Then, the ID cells are identified as ‘11′ (‘1′ from the first binary mask and ‘1′ form the second binary mask).

The RND cells that will be used to generate nonces (numbers used only once) are identified as ‘00′ (‘0′ from the first binary mask and ‘0′ form the second binary mask). The RND cells can be, for example, those cells changing their start-up values 50% of the measurements (in that case, the cells changing their start-up values, but not 50% of the times, are identified as ‘01′) . This is shown in the lower part of Figure 1a. In the example of Figure 1a, for simplicity, the RND cells are those changing once or more their start-up values (no ‘01′ cell is shown). RND cells extract the noise of the external operation conditions (temperature, voltage supply, etc.) as a source of entropy because, in them, noise of external conditions dominates over the minimum manufacturing process variability affecting their inverters.

At the end of the first registration phase, each sensor stores in a non-volatile memory the first and the second binary masks that indicate which cells are ID and which cells are RND. This information is not sensitive. The sensitive information is the start-up values, which are never stored in the non-volatile memory [20].

Although the start-up values from ID cells (ID response) have low bit flipping probability, the secret *K* is coded *(*$K_{coded})$ by an Error Correcting Code to cope with some bit flipping that can occur. Let us suppose that the secret to obfuscate is $K=\left[k_{1},\;\ldots ,k_{a}\right]$. Each bit is repeated r times so as to encode it with a repetition Error Correcting Code, $K_{coded}=\left[k_{11},\;\ldots ,k_{1r},\ldots ,k_{a1},\;\ldots ,k_{ar}\right]$. An ID response with a·r start-up values is obtained and the Helper Data (HD) are generated by XORing $K_{coded}$ and ID as $HD=ID⊕K_{coded}$. This is the Helper-Data generation stage of the Helper-Data algorithm, which is shown in Figure 1b. At the end of this stage, the sensor stores the Helper Data in the non-volatile memory.

The second stage of the Helper-Data algorithm is the secret reconstruction, which consists of the following steps illustrated in Figure 1c. An $\bar{\mathrm{ID}}$ response with a·r start-up values is obtained from the same ID cells employed in the Helper-Data generation by using the masks stored in the sensor. The $\bar{\mathrm{ID}}$ response is XORed with the stored Helper Data to obtain a bit string similar to $K_{coded}$, as follows:

(4) $${\bar{K}}_{coded}=\bar{\mathrm{ID}}⊕HD=\bar{\mathrm{ID}}⊕ID⊕K_{coded}.$$

Since $K_{coded}$, and *K*, are recovered from ${\bar{K}}_{coded}$ by using the decoder of a repetition Error Correcting Code, the probability of failure in reconstructing a bit of the secret *K* is equivalent to the probability that *r* bits of ${\bar{K}}_{coded}$ differ from $K_{coded}$ in *r/2* bits or more (caused by the flipping bits of $\bar{\mathrm{ID}}$). This can be calculated as $P_{total}$ in Equation (3) with n=r, t<r/2, and *p* estimated by the average fractional Hdist defined in (1) between $\bar{\mathrm{ID}}$ and ID responses. If the $\bar{\mathrm{ID}}$ response is truly generated by the ID cells used in the HD generation stage, *p* is very low (since ID cells are STB cells) and the probability of failure is also very low. Hence, a repetition Error Correcting Code works efficiently. Other works reported in the literature use more complex Error Correcting Codes such as BCH or Reed-Muller codes because they do not classify cells into STB and RND cells [11,21]. The processing to reconstruct the secret is shown in Figure 1c for the case of an 8-bit repetition Error Correcting Code.

If ID response is fully random, the secret is fully obfuscated and the Helper Data do not leak information [22]. To be random, a necessary condition of the ID response is to be debiased, as explained in [19], that is, the ID response should be uniform (should have the same number of ‘1′s and ‘0′s). This is why ID cells instead of STB cells are used to generate the Helper Data. If ID responses are uniform, the average fractional Hamming distance calculated as in (1) with the responses of different SRAMs (impostor distribution) tends to be 0.5.

Another way to evaluate the randomness of *k* ID responses with a·r bits is to calculate the minimum entropy as:

(5) $$H_{min}\;=\frac{1}{a\cdot r}\cdot \sum_{i=1}^{a\cdot r\;}log_{2}\left(p_{imax}\right).$$

where $p_{imax}$ is the maximum probability of the *i*-th bit taking logic value ‘0′ or ‘1′ in the *k* responses [9]. If $H_{min}$ is 1, the *k* ID responses are 100% independent and there are no correlations between the bits in different ID responses. This is the ideal situation for the case considered herein because, then, nodes with 100% independent ID responses can obfuscate a shared secret and store Helper Data that are quite independent and do not reveal information about the secret.

The minimum entropy can also be calculated for the RND cells to evaluate their randomness and their suitability as nonces. More details about how to measure the randomness of PUF responses can be seen in [9,22].

In the following, the Helper Data stored in the non-volatile memory of a sensor node D, which obfuscate a secret *K*, will be denoted as $HD_{K}^{D}=ID^{D}⊕\;K_{coded}$, where $ID^{D}$ is the physical identifier (ID response) of the sensor node D.

## 4. Novel Biometric Sensor Nodes Using QFM-Based Fingerprint Features

**Figure 2:**
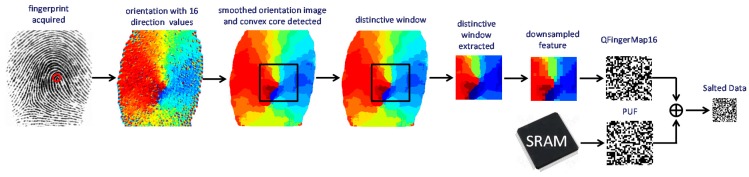
Salted Data generated with a QFM feature from a fingerprint image and a SRAM PUF response.

One of the most distinctive biometric traits is fingerprints. Fingerprint images are composed of ridges (depicted in dark in the fingerprint image from FVC 2002 DB1a shown in Figure 2) and valleys (depicted in white in Figure 2). Generally, fingerprint images are not compared directly at the verification step because there are variations between different captures. Instead, fingerprint images are processed to obtain distinctive features. According to the type of information extracted, there are three levels of fingerprint features: level 1 or global information such as orientation textures and singular points, level 2 or local information such as minutiae (ridge endings and ridge bifurcations), and level 3 or finer details (such as pores and scars). Distinctiveness is higher at higher levels of information, but also higher computational complexity is required. The use of texture-based features and singular points can offer better tradeoff between computational cost and recognition performance. Singular points are classified into delta points (situated where ridge lines intersect) and core points (located where ridge lines have maximum curvature), which in turn can be classified into convex and concave core points [15]. A convex core point is depicted with a red circle in the fingerprint image shown in Figure 2.

**Figure 3:**
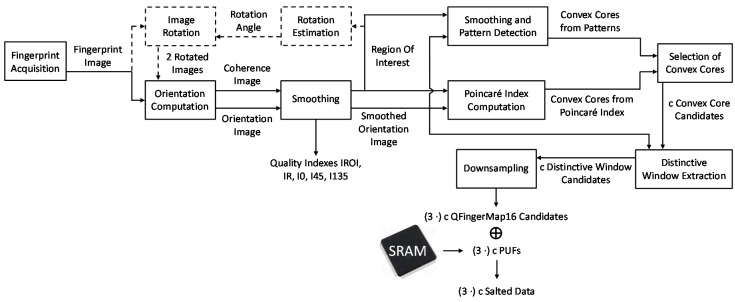
Extraction of the Salted Data based on QFMs and SRAM PUFs. Operations for fingerprint image rotation (illustrated in dashed lines) are employed when it is possible to acquire rotated fingerprint images. (3 × *c*) or *c* QFingerMap16 candidates are obtained if rotation operations are applied or not, respectively.

Among the texture-based approaches, well-known features are FingerCodes, Histograms of Oriented Gradients (HoG), Local Binary Patterns (LBP), and Local Directional Patterns (LDP) [23,24,25,26]. In order to develop novel biometric sensor nodes, this work proposes a novel texture-based feature named QFingerMap16 (QFM), which can be implemented with lower cost, as commented in the following. It is a refinement of the QFingerMap proposed in [27] to be more robust against noise so as to provide competitive results without complex image enhancement. The steps involved to work with QFM (orientation computation and quality estimation, alignment, feature extraction, and obfuscation) are illustrated in Figure 2 and Figure 3 and explained in the following.

### 4.1. Orientation Computation and Quality Estimation

The neighborhood operator described in [28] is employed to obtain the orientation image and to estimate the quality of the fingerprint image acquired from the user. It defines a fixed number, *N*, of reference orientations, *d_x_* with *x* = 1, …, *N*, and computes the standard deviation σ with the gray values of *n* neighbor pixels along each direction, with *n* at least (*N* + 2)/2. The value of σ along a direction d_x_ is compared to the value of σ along its orthogonal direction, and the pair of orthogonal directions (*d_x_*, *d_x+N/2_*) with the maximum difference of σ is selected to estimate the ridge orientation as follows:

(6) $$\Phi \left(i,j\right)=\{\begin{array}{c} \begin{array}{cc} d_{x} & \begin{array}{cc} if & \sigma \left(d_{x}\right)<\sigma \left(d_{x+N/2}\right) \end{array} \end{array}\\ \begin{array}{cc} d_{x+N/2} & \begin{array}{cc} if & \sigma \left(d_{x}\right)>\sigma \left(d_{x+N/2}\right) \end{array} \end{array}\\ \begin{array}{cc} \upsilon & \begin{array}{cc} if & \sigma \left(d_{x}\right)=\sigma \left(d_{x+N/2}\right) \end{array} \end{array} \end{array}.$$

where υ represents the orientation assigned to a homogeneous region, herein considered as 90°.

A neighborhood of 9 × 9 pixels (*n* = 9) centered at the analyzed pixel is considered and 16 possible reference orientations (from 0° to 180° in intervals of 11.25°) are evaluated (*N* = 16). In order to smooth the orientation image, the value exhibiting the highest frequency in a pixel-wise smoothing window of size 27 × 27 centered at each pixel is assigned to that pixel. The smoothing window size has been determined experimentally by considering different fingerprint databases [29].

The coherence of the dominant ridge orientation assigned to each pixel is qualified by a bit. The bit is 1 (orientation is coherent) if the difference of standard deviations between the selected orientation and its orthogonal direction is above a threshold. A threshold value of 10 provides good results according to experiments with different fingerprint databases. Otherwise, the bit is 0. The pixels whose coherence bit is 1 in the smoothed coherence image are considered to belong to the Region of Interest (ROI) of the fingerprint. A quality index IROI counts the smoothed coherence bits to evaluate if the fingerprint image has a ROI with enough size.

As proposed in [29], four more quality indexes, IR, I0, I45, and I135, are computed to evaluate if the fingerprint image has enough quality. The reliability of the orientation exhibiting the highest frequency in the smoothing window is qualified by another bit. The bit is 1 (orientation is reliable) if the frequency is above a threshold (chosen as 364, which is half of the pixels in the 27 × 27-pixel smoothing window). Otherwise, the bit is 0. A quality index IR counts the reliable bits to evaluate if the fingerprint has enough reliable pixels. The indexes, I0, I45, and I135, count, respectively, the reliable pixels inside the ROI whose dominant orientations are: (a) 0°, 11.25°, 157.5°, or 168.75° (that is, the orientations around 0°), (b) 22.5°, 33.75°, 45°, or 56.25° (orientations around 45°), and (c) 112.5°, 123.75°, 135°, or 146.25° (orientations around 135°). The dominant orientations around 90° are not counted since they are not in arch-class fingerprints.

The fingerprint image is evaluated as low quality if the index IROI or IR are low or the values of at least two of I0, I45, and I135 are low. This evaluation activates an alarm at the sensor node to make the user provide again his/her finger because such capture is not good to follow with the processing.

### 4.2. Alignment

Since the user puts his/her finger on the fingerprint sensor in a different way, translation and rotations should be taken into account to compare aligned fingerprints. The lightweight alignment solution employed herein is based on computing the possible convex core points in the image.

**Figure 4:**
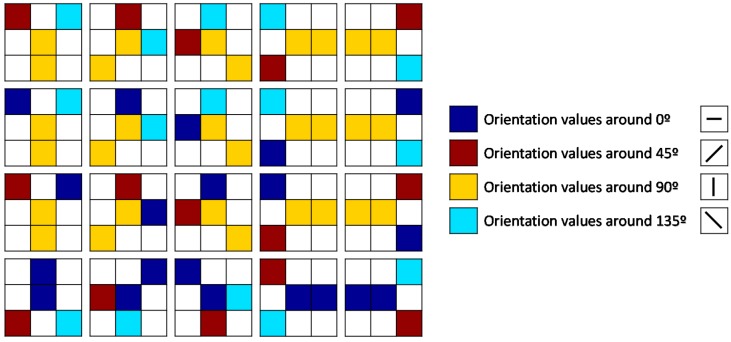
Patterns for the detection of convex cores.

**Figure 5:**
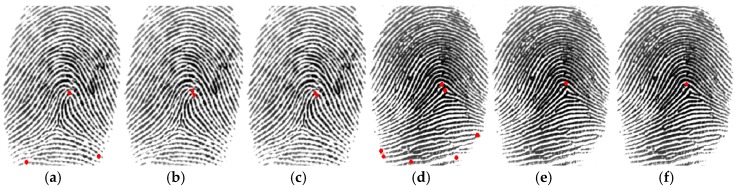
(**a**,**d**) Convex cores detected by Poincaré Index, (**b**,**e**) Convex cores detected by orientation patterns, (**c**,**f**) Convex cores detected by the proposed technique that combines Poincaré Index and orientation patterns. The fingerprint image employed in (**a**–**c**) is an example of non-arch fingerprint, and the fingerprint image employed in (**d**–**f**) is an example of arch fingerprint, both taken from FVC 2002 DB1a.

First, the Poincaré Index is calculated at each pixel (inside the ROI) of the smoothed orientation image commented above. Poincaré Index computation is the traditional method for the detection of singular points [15]. Second, the smoothed orientation image is smoothed again with the same pixel-wise smoothing window, it is converted to an orientation image with 4-orientation values (16-orientation values are converted to the dominant orientations around 0°, 45°, 90°, and 135°, as explained previously) and a set of 20 patterns with a kernel window of 3 × 3 pixels is also evaluated at each pixel. Figure 4 shows the 20 patterns employed. They consider the orientation values around 0°, 45°, 90°, and 135°. The last row corresponds to the patterns for the arch-class fingerprints (which do not include orientations around 90°). If a pattern of an arch-class fingerprint is found, the pixel location found by the pattern is selected as convex core candidate, since sometimes Poincaré index does not detect it. For a pixel that verifies one or more of the other 15 patterns, the pixel location found by Poincaré index which is closest to the location found by the pattern is selected, because the location found by Poincaré index is finer. Finally, the selected pixels are evaluated by the Moore-Neighbour tracing algorithm (modified by Jacob’s stopping criteria) [30] to find sets of neighbour points. Each set found is replaced by its geometric center pixel so as to reduce the number of convex core candidates. Usually, the number, *c*, of resulting convex cores is smaller than 6. Figure 5 illustrates an example of how the convex cores are detected by this technique for non-arch and arch fingerprint images.

Since the convex core candidates are used as reference points, the feature vectors are invariant to translations, but they change with rotations. If the finger can be placed rotated with respect to the vertical (due to the fingerprint image can be captured with rotations), a coarse rotation operation is applied to correct rotations to the fingerprint images. Firstly, the rotation angle of a fingerprint image is estimated based on the computation of the regression line of the ROI image. Then, if the fingerprint is found largely rotated to the right (more than 17° from the vertical, which is the middle between the 11.25° and 22.5°), a rotation of 11.25° to the left, and another rotation of 11.25° to the left are applied so as to move the fingerprint image towards the vertical. On the other side, if the fingerprint is found largely rotated to the left of the vertical, the rotations of 11.25° are applied to the right. If the fingerprint is found to be slightly rotated, a rotation of 11.25° to the left and another rotation of 11.25° to the right are applied to the fingerprint image.

### 4.3. Feature Extraction

Distinctive information is extracted from the smoothed orientations, in the *c* windows of *w* × *w* pixels centered at the *c* selected convex cores. The size of the window adequate for the recognition process depends on the size of the fingerprint captures. For most sensors, which capture images of approximately 300 × 300 pixels, a suitable window size is 128 × 128. It offers a good trade-off between information extracted (smaller windows lose information) and enough pixels inside the ROI (larger windows are more probably outside the ROI).

In order to compact information, not all the pixels in a distinctive window are considered but a down-sampling by a factor of *m* is applied (one pixel is considered out of *m* consecutive pixels). After experiments with different fingerprint databases, the most suitable down-sampling factor was found to be 8. Hence, the QFM-based fingerprint feature contains *w* × *w*/*m* = 128 × 128/8 = 16 × 16 orientations, which means 1024 bits (the 16 possible orientations are coded by 4 bits). If the fingerprints can be acquired with rotations, three smoothed orientation images are evaluated per fingerprint capture, analyzing *c* windows from each of them for *c* convex core candidates. Hence, the feature vector of one capture can be formed by 3 × *c* QFM-based features.

### 4.4. A Low-Cost Solution to Obfuscate Sensitive Fingerprint Information at Sensor Nodes

Since the QFM-based features contain sensitive information about users, they should not be stored or transmitted without protection. Taking advantage that they are binary strings, our proposal is to obfuscate them with the PUF responses of the sensor node where the user has to be recognized. A kind of biometric salting is used for this purpose where the PUF response is used as salt.

Biometric Salted Data are generated in a registration (enrollment) phase whenever an accredited individual needs using the sensor, in a secure environment. The user is required to place his/her fingers in the fingerprint sensor of the sensor node and the QFM-based features are extracted. The quality control performed as described in Section 4.1 allows interacting with the user to acquire good captures. If several captures, *t*, of several fingers, *f*, are captured, the feature information obtained is $\left\{\mathrm{QFM}\right\}=\left\{QFM_{ijk}\right\}$ with i=1, …,t; j=1, …,f; and k=1, …,c; *c* being the total number of QFM features extracted from each capture.

A total of t·f·c ID responses are obtained from the SRAM PUF as described in Section 3.2 to generate a set of Salted Data as $\left\{SD\right\}=\left\{ID_{x}⊕\;QFM_{x}\right\},\;x=1\ldots ,t\cdot f\cdot c$. For example, for two fingers per user, three samples per finger, and two QFM-based features per sample, the SD have 1.5 KBytes per user. If ID responses are fully random, the biometric information is fully obfuscated [18,19,31]. Nothing is revealed about who user is registered at each node. The SD generated at node D with user *X* are denoted as $SD_{X}^{D}$. The same t·f·c ID responses are employed to obfuscate t·f·c secrets, $K_{x}$, encoded with the Error Correcting Code as described in Section 2.2, and generate other $\left\{HD\right\}=\left\{ID_{x}⊕\;Kcoded_{x}\right\}$, which are denoted as $\left\{HD_{K}^{D}\right\}$ for node D. Each sensor node, D, stores $\left\{SD_{X}^{D}\right\}$ and $\left\{HD_{K}^{D}\right\}$ in its non-volatile memory.

The second phase of secret reconstruction is carried out whenever the secrets are needed. It consists of the following steps for a given sensor node D and a user X. A set of t·f·c responses are obtained from the same ID cells employed in the registration phase. They are XORed with the stored $\left\{HD_{K}^{D}\right\}$ to obtain bit strings similar to $Kcoded_{x}$, which are decoded to recover the original t·f·c secrets, $K_{x}$, and the original t·f·c ID responses employed at registration. From the original t·f·c ID responses XORed with the Salted Data $SD_{X}^{D}$, the original $\left\{QFM^{X}\right\}=\left\{QFM_{ijk}^{X}\right\}$ of user X are recovered. The user is required to place his/her fingers in the fingerprint sensor of the sensor node and the features ${\bar{QFM}}_{ljm}^{X}$ are extracted, with l=1, …,q, the number of samples captured at matching; j=1, …,f; and m=1, …d; *d* being the total numbers of QFM-based features extracted from each capture.

The matching score that evaluates if the user is or not recognized is calculated as follows:

(7) $$D\left(\left\{QFM_{ijk}^{X}\right\},\left\{{\bar{QFM}}_{ljm}^{X}\right\}\right)=\frac{1}{1024\cdot f}\cdot \sum_{j=1}^{f}\left\{\underset{\begin{array}{c} i=1,\ldots ,t\\ l=1,\ldots ,\;q \end{array}}{\min}\left[\underset{\begin{array}{c} k=1,\ldots ,c\\ m=1,\ldots ,\;d \end{array}}{\min}\left[Hdist\left(QFM_{ijk}^{X},{\bar{QFM}}_{ljm}^{X}\right)\right]\right]\right\}.$$

A threshold is fixed in the sensor nodes so that if the matching score is smaller than the threshold the user is accepted while he/she is rejected otherwise. The threshold value should be adjusted to provide a good trade-off between false rejected users and false accepted users depending on the application.

## 5. Lightweight Protocol to Authenticate Sensor Nodes by Dual-Factor

Let us consider a sensor network with sensors that are able to exchange information and transmit their readings to a base station. Taking into account that sensor nodes are resource-constrained in terms of processing, memory and power consumption, the communication protocol should be lightweight. The base station has more capabilities to process the sensed data and to communicate them to other networks such as Internet. Our focus is on security at sensor nodes. Base station security is outside the scope of this paper.

The proposed protocol is able to exploit the dual-factor authentication of the sensors presented in Section 3 and Section 4. It contemplates a registration phase in a secure environment. As described in Section 4.4, each sensor node, D, stores Salted Data, $\left\{SD_{X}^{D}\right\}=\left\{ID_{x}^{D}⊕\;QFM_{x}^{X}\right\}$, and Helper Data, $\left\{HD_{K}^{D}\right\}=\left\{ID_{x}^{D}⊕\;Kcoded_{x}\right\}$, in its non-volatile memory, for the legitimate user X who can authorize the node to transmit information. One of the x secrets, $\left\{K_{x}\right\}$, encoded with the Error Correcting Code, is used as the global master secret of the network, $K_{0}$. If the $\left\{ID_{x}^{D}\right\}$ are fully random, Helper Data do not reveal nothing about the master secret and the legitimate user.

**Figure 6:**
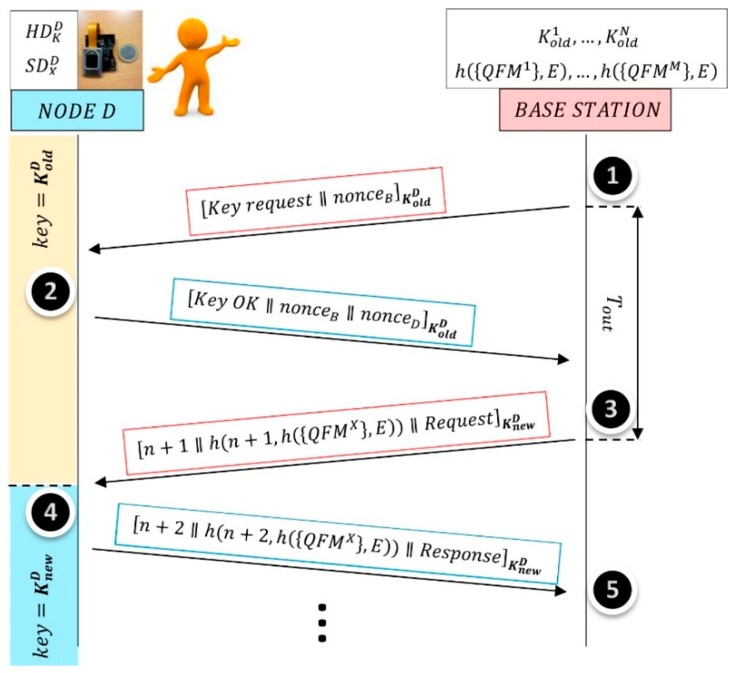
Proposed lightweight protocol.

Figure 6 illustrates the proposed protocol between a sensor node D and the base station. The notation used to describe the operations is the following:

- [a∥b] represents the plaintext data “*a*” concatenated with the plaintext data “*b*”.
- ${\left[c\right]}_{K}$ represents the plaintext data “*c*” encrypted by the key “*K*” using, for example, the block cipher AES (Advanced Encryption Standard). AES was approved in 2001 as a US federal standard (FIPS PUB 197) and then, it was included in the ISO/IEC 18033-3 standard. Hence, AES is the dominant symmetric-key algorithm in many commercial applications and it is included as hardware module in many sensor node microcontrollers.

The messages are authenticated by a Message Authentication Code, MAC. The most popular approach in practice is to use a block cipher such as AES in Cipher Block Chaining (CBC) mode according to NIST 800-38A. This is not illustrated in Figure 6 to simplify the scheme. The notation used to describe the message exchanges is as follows:

- “n” represents a randomly initialized index which increases by 1 both in the sensor and the base station simultaneously after each message exchange is successfully completed.
- ${\mathrm{nonce}}_{B}$ and $nonce_{D}$ represents, respectively, the random seed generated by the base station and the sensor, which are used to derive the new key $K_{new}$ from the old key $K_{old}$.
- *h* (*x*,*E*) refers to a cryptographic hash function approved by NIST and “*E*” is the identifier of the network.

A message time-out, $T_{out}$, is established for each message pair exchanged. That is, each message sent needs a response in a specific time. If the response is not received before the time-out, the message is retransmitted again.

When the base station requires sensor node D to communicate sensed data, a mutual authentication is performed previously. In order to ensure that symmetric keys are used only once to increase security, a session key, $K_{new}^{D}$, is derived from the previous session key, $K_{old}^{D}$. The first time a communication is initiated with a node, the shared secret key is used, $K_{old}^{D}=K_{0}$. Following NIST recommendations [32], a Key Derivation Function, KDF, is applied to $K_{old}^{D}$ and the random seeds (nonces) interchanged at steps 1 and 2, to obtain $K_{new}^{D}=KDF\left({\mathrm{nonce}}_{B},\;{\mathrm{nonce}}_{D},K_{old}^{D}\right)$. 

At step 2, sensor node D reads the start-up values of its SRAM cells and obtains the responses $\left\{{\bar{ID}}_{x}^{D}\right\}$. With them and $\left\{HD_{K}^{D}\right\}$ the sensor node is able to recover the $K_{old}^{D}$ ($K_{0}$ in the first session) and the $\left\{ID_{x}^{D}\right\}$. With them and $\left\{SD_{X}^{D}\right\}$, the sensor node is able to recover the $\left\{QFM_{x}^{X}\right\}$. Only the trusted node is able to recover the $K_{old}^{D}$ and the biometric data of its user. The start-up values of a set of RND cells are also read to generate the $nonce_{D}$.

At step 4, sensor node D checks that the new key has been updated by the base station at step 3. Hence, it changes $HD_{Ko}^{D}=\left\{ID_{Ko}^{D}⊕\;Kcoded_{0}\right\}$ by $HD_{knew}^{D}=\left\{ID_{Ko}^{D}⊕\;Kcoded_{new}\right\}$, which will be used in the next session as the initial symmetric key to derive another session key. Sensor node D is able to decrypt the message sent by the base station and to verify the authenticity of base station because it knows, not only $K_{old}^{D}$ but also $h\left(\left\{QFM^{X}\right\},E\right)$, which is only stored at base station. Sensor node requires the user to place his/her fingers in the fingerprint sensor and the features $\left\{{\bar{QFM}}^{X}\right\}$ are extracted and compared with $\left\{QFM^{X}\right\}$ locally. If the user is legitimated as *X*, the sensor node sends the requested data.

At step 5, the base station checks the dual-factor authenticity of sensor node D and receives the data. Once the session finished, base station also stores $K_{new}^{D}$ as $K_{old}^{D}$ to use it in next sessions with sensor node D. Base station stores the *N* keys to communicate with the *N* nodes. 

Biometric data cannot be obtained from the interchanged messages because they are protected by a hash, which is a one-way function and, besides the result is encrypted together with a nonce, by a fresh key. The message indexes avoid replay attacks because they are used only once for each communication. The base station stores the *M* $h\left(\left\{QFM^{X}\right\},E\right)$ to cope with the *M* individuals in the network, but even if all $h\left(\left\{QFM^{X}\right\},E\right)$ are leaked, they don’t reveal any sensitive information related to the individuals (they are like pseudonymous) and they are isolated to this network. This way if a network is successfully attacked it will not affect other networks and users.

This protocol allows the highest level of security in electronic authentication of sensor nodes as defined by the digital identity guidelines of the NIST SP 800-63 [33], since it is based on the device authentication and also on the authentication of the individual which uses it.

## 6. Experimental Results and Discussion

Since the proposed protocol provides confidentiality, integrity, and freshness (with derived keys and nonces), remote attacks are reduced. Experimental results in Section 6.1 and Section 6.2 show that the proposed sensors reduce counterfeiting, impersonation, and non-repudiation attacks (from fake sensors or malicious users). Section 6.1 also shows that information leakage from Helper Data is negligible. Section 6.3 illustrates implementation results in low-cost hardware. Experimental results in Section 6.4 show that even accessing the sensor or stealing it, the attacker has a small probability to be considered as legitimate. Section 6.4 also shows that information leakage from Salted Data is negligible and that entropy of nonces is enough for the key derivation function.

### 6.1. Trusted Sensor Nodes Based on SRAM PUFs

The first question addressed experimentally was to evaluate if sensor nodes can be identified unequivocally by their SRAM PUFs. For that purpose, the communication module of the node was selected as a good candidate where to find SRAM PUFs, because, in order to save power budget, communication module is better powered down by the main processor of the node at times whenever no communication is carried out. Whenever communication is needed and the module is powered up, the start-up values of the SRAM are obtained. Since many sensor nodes use wireless communications based on Bluetooth Low Energy (BLE), BLE chips were analyzed. Among the BLE system on chips (SoCs) commercially available, the CC2541 from Texas Instruments was selected. The CC2541 contains an Intel 8051 microcontroller capable to access 256-KB of in-system-programmable flash and 8-KB SRAM through a memory arbitrator block. That SRAM was evaluated in 10 BLE chips, taking 56,000 bits per SRAM (7000 bytes) and measurement.

A specific firmware was developed to carry out the first registration phase of the BLE chip. It powered down and up the SRAM 20 times and compared the start-up values obtained to classify the SRAM cells into STB and RND cells and to select the ID cells from the STB cells that provided debiased values (as described in Section 3.2). The end of the first registration phase is that the binary masks that identify the ID and the RND cells are stored in the non-volatile memory of the BLE chip. This information is not sensitive. Since the first registration phase is carried out before the sensor node is deployed, there is no problem of power consumption at this phase. Once the ID and RND cells are identified and stored, the subsequent key reconstruction stages need only one powered-up as well as the subsequent registration stages whenever the session keys are derived. After registration, Helper Data obtained from each chip are stored, $\left\{HD_{K}^{D}\right\}=\left\{ID_{x}^{D}⊕\;Kcoded_{x}\right\}$, and the responses $\left\{ID_{x}^{D}\right\}$ are not stored anywhere.

The minimum number of ID cells found in the BLE chips was 14,758. Hence, CC2541 was able to generate at least 14 ID responses with 1024 bits, which can obfuscate 14 secret keys of 128 bits encoded with an 8-bit Error Correcting Code (and generate the corresponding Helper Data) as well as obfuscate two QFM-based features per sample, three samples per finger, and two fingers per user (and generate the corresponding Salted Data). For the analysis carried out herein, these responses were enough. If more ID cells are needed (to obfuscate more QFM-based features per sample), other debiasing algorithms, as reported in [19], can be employed instead of pair-output von Neumann or 2O-VN debiasing algorithm employed herein. The minimum number of STB cells found in the BLE chips was 41,213 and this debiasing algorithm reduces them to 14,758 ID cells.

**Figure 7:**
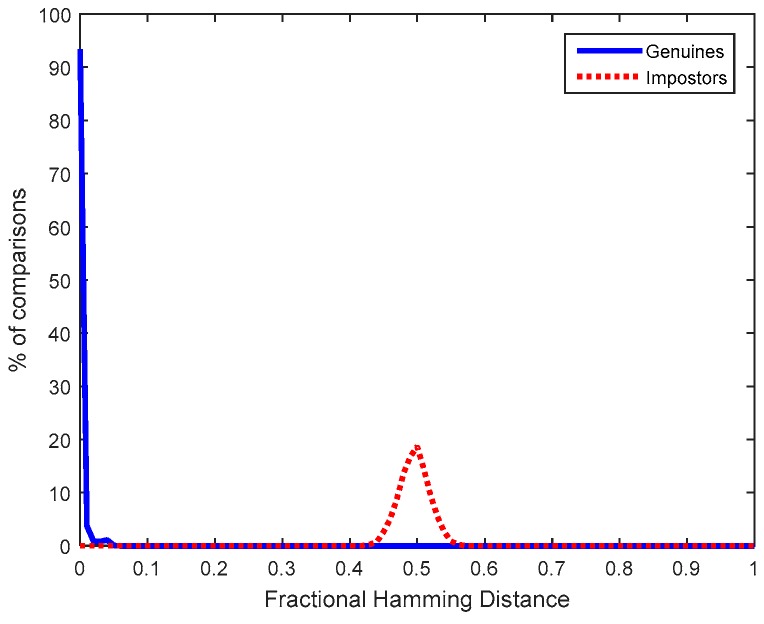
Fractional Hamming Distances obtained from SRAM PUFs.

Once registered, other 20 measurements were taken to evaluate the difference between the new ID responses obtained, $\left\{{\bar{ID}}_{x}^{D}\right\}$, and the ID responses considered for the Helper-Data generation, $\left\{ID_{x}^{D}\right\}$. Figure 7 illustrates at the left part the distribution of fractional Hamming distances (Hdist) obtained from the comparisons of $\left\{{\bar{ID}}_{x}^{D}\right\}$ and $\left\{ID_{x}^{D}\right\}$, for any chip *D* (*D* = 1,…, 10) and any response (*i* = 1,…, 14), which corresponds to the genuine population of ID responses. The average fractional Hdist, calculated as in (1), is 0.0063.

Considering that the secret *K* is encoded into $K_{coded}$ at the Helper-Data generation stage and decoded from ${\bar{K}}_{coded}$ at the reconstruction stage by using an 8-bit repetition Error Correcting Code, the probability of failure in reconstructing a bit of the secret *K* (as was described in Section 3.2) is equivalent to the probability that 8 bits of ${\bar{K}}_{coded}$ differ from $K_{coded}$ in 4 bits or more. Applying Equation (3) with n=8, t=3, and *p* estimated as 0.0063 (the average fractional Hdist of the genuine responses measured experimentally as commented above), the probability of failure in reconstructing a bit of the secret *K* is 1.08 × 10^−7^ (Equation (3) is evaluated as 1-binocdf(3, 8, 0.0063) in Matlab). Therefore, the probability to find some error in a 128-bit secret is 1.38 × 10^−5^ (1-binocdf (0, 128, 1.08 × 10^−7^)). If lower bit error rate is required, the codeword length of the repetition ECC can be increased. For example, considering a 16-bit ECC, the probability of failure in reconstructing a bit of the secret *K* is 3.05 × 10^−14^ (1-binocdf (7, 16, 0.0063)) and the probability to find some error in a 128-bit secret is 3.91 × 10^−12^ (1-binocdf (0, 128, 3.05 × 10^−14^)).

The above commented results correspond to nominal operation conditions and to the model based on Equations (1)–(3) described in Section 3.2. However, the reliability of a PUF is usually evaluated under different environmental conditions. Bit flipping in SRAM PUFs is mostly affected by changes in ramp-up time (i.e., the time to reach power supply voltage value after power-up) and temperature [8,9]. Supposing that the ramp-up time cannot be modified (because the on-chip voltage regulator cannot be tampered), the robustness of the key reconstruction stage using an 8-bit repetition ECC against changes in operation temperature was evaluated. A specific firmware was developed to carry out the key reconstruction stage and five CC2541 chips were evaluated more exhaustively at 5 °C, 25 °C, and 75 °C with 100 measurements for each temperature and 4096 secret bits reconstructed for each chip by using the 8-bit repetition ECC. In total, for the three temperatures and the 100 measurements, 6,144,000 bits were reconstructed for the 5 chips. For all the measurements, only 20 bits were not reconstructed correctly (corresponding to 4 bits for 5 measurements at 75 °C). That means a failure rate of 20/6,144,000 = 3.26 × 10^−6^. This can be acceptable since an error rate of 10^−6^ is considered by many authors as a conservative value that fulfils the requirements of most of typical security applications [11,21]. Therefore, secret keys of 128 bits (that is, for example, the key length for the standard cipher AES) can be recovered by the 1024-bit ID responses of the genuine sensor node with high probability. Hence, the QFM-based features employed at registration can also be recovered.

The distribution of fractional Hamming distances (Hdist) obtained from the comparisons of $\left\{{\bar{ID}}_{i}^{A}\right\}$ and $\left\{ID_{j}^{B}\right\}$, for any pair of chips (A≠B, ∀ i,j) or any pair of responses (i≠j, ∀ A,B), which corresponds to the impostor population of ID responses is illustrated at the right of Figure 7. The average fractional Hdist, calculated as in (1), is 0.5004. Hence, the bit flipping probability of impostor responses can be approximated by 50.04%, as described in Section 3.2. The probability of having the same bit value in two different ID responses was like tossing a coin. No correlation was found among the start-up values of different ID responses (they were independent), from the same or different SRAMs. Hence, no correlation is found between Helper Data $\left\{ID_{x}^{D}⊕\;Kcoded_{x}\right\}$ stored in the same sensor node as well as between Helper Data in different sensor nodes. A secret key obfuscated by one ID response cannot be recovered by a different ID response.

The minimum entropy of the impostor responses, calculated as in (5), is 91.92%. This means that in a 1024-bit response, there are 941.26 independent bits and the secret is almost fully obfuscated because the response is almost fully random.

### 6.2. Dual-Factor Authenticated Sensor Nodes

Another question addressed was to evaluate if a sensor node can be identified unequivocally by its SRAM PUFs and its user, that is, if the sensor node D used by the individual X is the unique node that can obtain the Helper Data $\left\{HD_{K}^{D}\right\}=\left\{ID_{x}^{D}⊕\;Kcoded_{x}\right\}$ and $\left\{SD_{X}^{D}\right\}=\left\{ID_{x}^{D}⊕\;QFM_{x}^{X}\right\}$ or similar at registration. Any other node used by another individual cannot be registered with similar Helper and Salted Data. Hence, the node D used by the individual X is the unique node that can provide the required response using the Helper Data $\left\{HD_{K}^{D}\right\}=\left\{ID_{x}^{D}⊕\;Kcoded_{x}\right\}$ and the Salted Data $\left\{SD_{X}^{D}\right\}=\left\{ID_{x}^{D}⊕\;QFM_{x}^{X}\right\}$ stored in its non-volatile memory.

**Figure 8:**
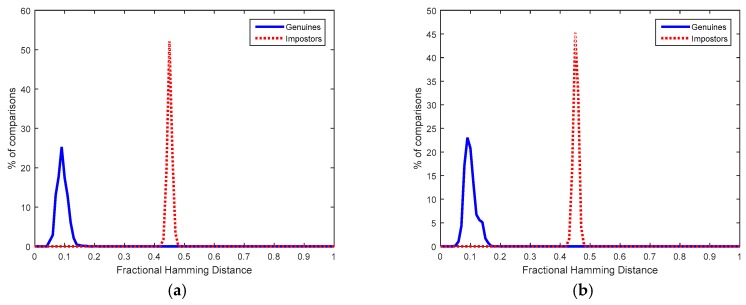
Fractional Hamming Distances obtained from Helper Data associated to BLE PUFs and individuals whose fingerprints were taken from: (**a**) FVC 2000 DB2a and (**b**) FVC 2002 DB1a.

Figure 8a,b illustrate at the left part the genuine distribution of matching scores obtained when the node D used by the individual X (using two fingers and three samples per finger at registration and two fingers and two samples per finger at verification) employs its associated Helper and Salted Data (which means calculation of Equation (7) with the original $QFM_{x}^{X}$ of user X correctly recovered).

The impostor distributions shown at the right of Figure 8a,b represent the matching scores obtained when the Helper and Salted Data of one node used by one individual are used by a different node and individual (which means calculation of Equation (7) with QFM-based features incorrectly recovered). Although these impostor distributions are not centered exactly in 0.5 because the bits which compose the QFM-based features are not fully independent, authentication can be carried out without error because the maximum Hdist of the genuine distribution is smaller than the minimum Hdist of the impostor distribution.

In order to obtain QFM-based features from fingerprints, public and standard databases were employed. In this case, the FVC 2000 DB2a (which includes captures from a capacitive sensor) and the FVC 2002 DB1a (with fingerprints acquired by an optical sensor) were considered. Both fingerprint databases are composed of 800 fingerprint images (100 different fingers from 100 different individuals and eight samples per finger). A virtual database was created with real fingerprints from these databases associated with real SRAM PUFs from measurements of the commercial CC2541 BLE chips. The impostor distributions were determined by the three first samples of a finger (taken as registered data) compared to the two first samples of the rest of individuals (taken as data to match). The genuine distributions were determined by all the possible combinations of three samples (taken as registered data) compared to the rest of combinations of one or two samples (taken as data to match) for the same individual. Each individual is assumed to be recognized by using two fingers (taken consecutively from the database).

Since the steps of the solution proposed to obtain the QFM-based features allow estimating the quality of the fingerprint sample acquired, the sample is not considered for recognition if it is estimated as a bad-quality acquisition. In addition, two QFM-based features are compared if the recognition area is sufficient. If such fingerprints are not considered, the number of different impostor recognition attempts is: 1315 and 1987, for FVC 2000 DB2a and FVC 2002 DB1a, respectively. The number of different genuine recognition attempts is 2450 and 5160, for FVC 2000 DB2a and FVC 2002 DB1a, respectively. When a QFM is obtained with a recognition area smaller than 1024 bits, padding is applied to reach the fixed length of 1024 bits.

### 6.3. Implementation Results

**Figure 9:**
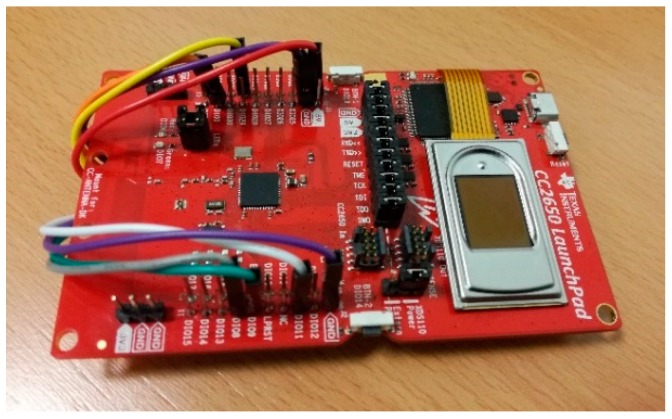
A low-cost sensor node prototype: CC2650 connected to the FPC1011F3 fingerprint sensor.

Besides the specific firmware developed to generate ID responses from the BLE chip in the sensor node, the proposed fingerprint recognition solution based on the QFM features was programmed in C code, using fixed-point arithmetic since none of the steps requires floating point operations. The results shown correspond to 152 × 200-pixel fingerprint images with 256 grayscale values because demonstrators were developed using the capacitive sensor FPC1011F3 [34], which is suitable for low-cost sensor nodes. This fingerprint sensor was connected through the Serial Peripheral Interface (SPI) to a Texas Instruments CC2650 LaunchPAD and to a Texas Instruments Tiva C Series TM4C123G LaunchPad in order to implement the proposal into 32-bit ARM Cortex-M3 and Cortex-M4 processors for microcontroller units, respectively, as shown in Figure 9. No rotation is applied to the captures because the sensor FPC1011F3 guides the user to place the finger always with a similar vertical orientation.

**Figure 10:**
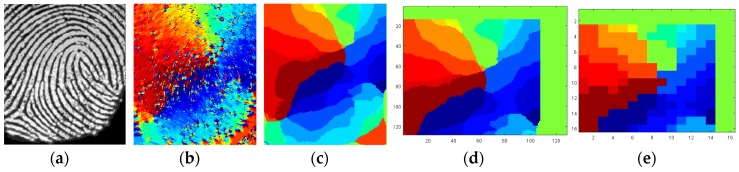
Results from the C code in the prototype: (**a**) Fingerprint acquired, (**b**) Orientation image with 16 directions, (**c**) Smoothed orientation image, (**d**) One of the two distinctive windows, (**e**) The corresponding QFingerMap16 feature.

The results at the successive steps of the processing are shown in Figure 10. They were verified by comparing them with the results of the proposed recognition solution programmed in Matlab and executed in a PC. The experimental results in Figure 10 illustrate how the proposal is robust to some noise level in the fingerprint acquisition.

A first advantage of the proposed fingerprint recognition solution is the small size of the fingerprint feature. The QFM-based feature requires 128 bytes while other texture-based features require 512 bytes (in the case of FingerCodes considering 64 sectors and 8 Gabor filters [26]) and 1456 bytes (in the case of LDPs considering a 56-bin histogram for each sub-window, 16 subwindows, and 85 × 85 pixels per sub-window [26]). It is noted that for minutia-based solutions the performance is compromised for features with less than 500 bytes, as reported in [35] where 1000 bytes are employed (by assuming 80 minutiae and 12.5 bytes for each minutia). The minutia-based feature of the embedded fingerprint recognition solution reported in [36] employs an average size of 512 bytes.

The results of the implementation of our proposal in ARM Cortex-M3 and M4 microcontrollers are shown in the first two rows of Table 1. The other rows in Table 1 correspond to other embedded fingerprint recognition solutions reported in the literature. Most of them are software implementations executed into microprocessor units, working at moderate frequencies (a few hundreds of MHz). Since most of the referenced proposals do not provide power consumption results, the values included in the last column of Table 1 were obtained from the datasheets or works focused on power consumption studies of the implementation platforms considered [42,43,44,45,46,47,48]. It can be seen how the proposed solution consumes very low dynamic power, maintaining real-time processing for the whole recognition process. Hence, it is very suitable for implementing fingerprint recognition locally in low-cost sensor nodes. The other proposals in the literature employ minutia-based algorithms, which are much more complex than the algorithm we propose. Therefore, even implementing them in complex platforms (with higher power consumption), the resulting processing times are longer.

### 6.4. Security and Privacy

Let us consider that an attacker accesses the sensor or steals it physically. To evaluate his/her probability of success, the same genuine and impostor distributions described in Section 6.2 were considered. Table 2 includes the recognition results in terms of Equal Error Rate (EER), which is the error rate where False Matching Rate (FMR) is equal to False Non-Matching Rate (FNMR).

Hence, even stealing the sensor node, the probability of successful impersonation attacks is low. Concerning biometric recognition, there is always a trade-off between usability of the solution and recognition performance. If only one instead of two fingers and fewer samples per finger are required from the user, the EER and the usability increase.

Table 3 compares these results of the proposed solution with other proposals reported in the literature to implement biometric recognition in wearable sensor nodes. In most of proposals in the literature, the wearables are employed for biometric data acquisition but the recognition algorithm is performed outside the wearable because the algorithms are computationally complex [49,50,51,52]. The fingerprint-based solutions implemented as software in embedded devices (like [36]) have a high power consumption or response time that is not suitable for many sensors, as was shown in Table 1. In order to reduce power consumption and response time, other authors design dedicated hardware for fingerprint recognition [53,54,55]. Our proposal can be implemented inside the low-cost wearable sensor node, achieving good distinctiveness without requiring dedicated hardware.

**Figure 11:**
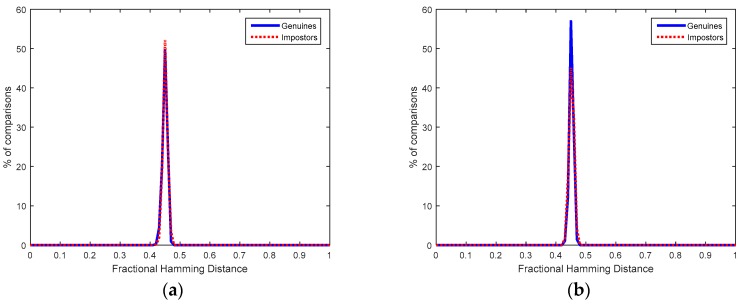
Unlinkability evaluation for 2 fingers and 3 samples in enrollment and 2 fingers and 2 samples in verification for: (**a**) FVC 2000 DB2a and (**b**) FVC 2002 DB1a.

Another question concerning privacy is to evaluate if an attacker could know by inspection of the non-volatile memories of the sensor nodes if the same individual is legitimated to use them or not. For that purpose, the biometric Salted Data generated from different samples of fingerprints from an individual who is registered at different nodes are compared with the Salted Data generated from different individuals registered at different nodes. The distributions obtained for the multi-biometric approach based on two fingers and three samples in enrollment and two fingers and two samples in matching are shown in Figure 11. Both distributions coincide because they overlap extensively. Therefore, if an attacker accesses two biometric Salted Data in two different sensor nodes, he/she is not able to discover if they have been generated by the same or different individuals, which is known as unlinkability. For this evaluation, as in the Section 6.2, a virtual database with real fingerprints from fingerprint databases (FVC 2000 DB2a and FVC 2002 DB1a) were associated with real 1024-bit ID responses extracted from the SRAM PUFs in the CC2541 chips.

Another advantage of the proposal herein is the revocability (also known as renewability or cancellable data) because the Helper and Salted Data can be replaced (by using other PUF responses) in case of information leakage.

Finally, another question addressed was the capability of the RND cells in the SRAM PUF to generate the nonces employed in the communication protocol to derive the session keys. The minimum number of RND cells found in the BLE chips was 265 (these RND cells were selected in the first registration phase as those which changed 10 times their start-up values in the 20 measurements). Hence, CC2541 was able to generate nonces with 128 bits. The Hamming weight of the nonces tended to be 0.5, that is, the number of ‘1’s and ‘0’s tended to be the same, which is interesting for the key derivation function of the protocol.

**Figure 12:**
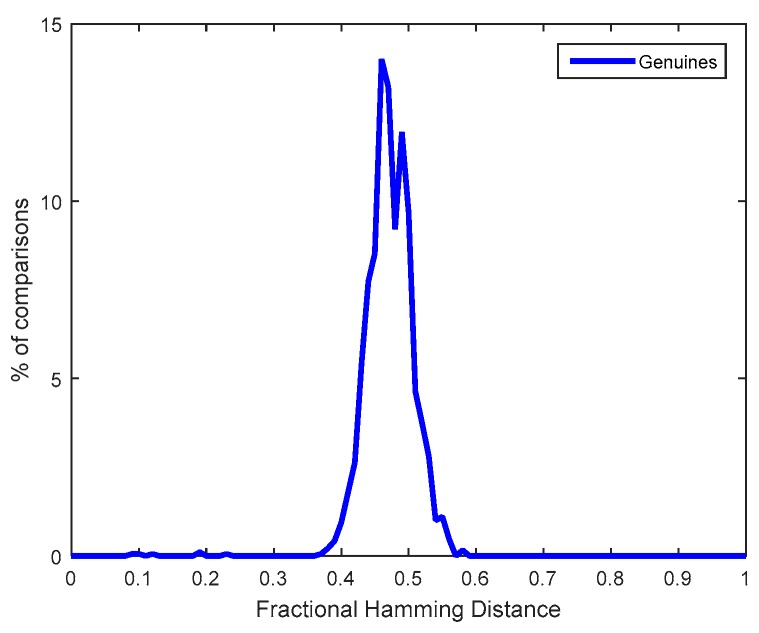
Fractional Hamming Distances obtained for sequences generated by RND cells.

Figure 12 illustrates the distribution of fractional Hamming distances (Hdist) obtained from the comparisons of pairs of RND sequences generated by the same RND cells at different start-ups (considering the 10 chips). The average fractional Hdist, calculated as in (1), is 0.48 and the dispersion of the distribution is considerable, which means that the start-up values of several RND cells do not behave as tossing a coin and, hence, they are highly but not fully entropic (if RND cells had not been selected in the first registration phase, their entropy would have been much lower, as explained in [9]). The minimum entropy calculated as in Equation (5) with different number of measurements (from 1 to 100 start-ups) from the RND cells of one of the devices was 75%, which is enough for nonces. Hence, the RND cells are suitable to generate the nonces for the key derivation function.

## 7. Conclusions

Security at sensor nodes is increased by means of a dual-factor authentication based on the unique physical identity of the node and the physical presence of the legitimate user. SRAM PUFs extracted from commercial BLE communication modules allow obfuscation of sensitive data into non-sensitive Helper and Salted Data. In the one side, only the trusted sensor node is able to recover from the Helper Data the 128-bit secret keys used to provide confidentiality and integrity to the transmitted messages as well as to derive fresh session keys. In the other side, only with the physical presence of the legitimate user, the trusted sensor node is able to recover from the Salted Data the secret set of 1024-bit fingerprint features whose hash is transmitted to provide non-repudiation. The proposed lightweight communication protocol reduces the possibilities of remote attacks to recover the sensitive information. Results from the BLE PUFs and the fingerprints of the public and standard databases FVC 2000 DB2a and FVC 2002 DB1a show that even stealing the sensor node, the probability of successful impersonation attacks is low. Besides, if an attacker accesses the Salted Data in two different sensor nodes, he/she is not able to discover if they have been generated by the same or different individuals because they show high unlinkability. Implementation results show that the fingerprint recognition of the user (which is computationally more costly than the hardware recognition of the PUF) can be executed as software in microcontroller units, featuring power consumptions of only a few milliwatts, feature extraction times of only a few seconds, and matching times below the millisecond. Hence, hardware required is so lightweight that the proposed security solution can be fully implemented in the sensor node.

## Figures and Tables

**Table 1:** Software implementations of fingerprint recognition solutions in embedded systems.

Proposal	Platform	Freq. (MHz)	Extraction Time (s)	Matching Time (s)	Power (mW)
QFM	ARM Cortex-M3	120	2.21	2.62 × 10^−4^	1.32
QFM	ARM Cortex-M4	204	1.12	1.46 × 10^−4^	2.50
Minutiae [37]	ARM922T	200	15.74	280.01	180
Minutiae [38]	NIOS	33	55.88	0.15	>200
Minutiae [39]	Microblaze	100	2.65	140.04	>269
Minutiae [40]	Strong ARM SA-1110	206	0.56	0.01	<400
Minutiae [41]	LEON2 without FPU	50	157	51	500
Minutiae [36]	LEON2 with FPU	50	9.04	0.067	500

**Table 2:** Recognition results by rejecting captures evaluated with bad quality.

DB		FVC 2000 DB2a	FVC 2002 DB1a
3 samples in enrollment. 2 samples in verification. 2 fingers	EER (%)	0.04	0.22

**Table 3:** Comparison of proposals for wearable sensor nodes.

Proposal	No. Individuals	Error Rate (%)
Gait [49]	59	EER = 2.6
Electrocardiogram [50]	28	FMR = 5.2, FNMR = 1.9
Touch Behavioral and Voice [51]	32	EER = 9–16.6 for touch behavioral
		EER = 4.88 for voice
Human Body Communication [52]	10	EER = 7.06
Fingerprint (one finger) [36]	10	FMR < 0.1, FNMR = 1
Fingerprint (one finger) [53]	96	FMR = 1.07, FNMR = 8.33
Fingerprint (one finger) [54]	100	EER = 8 (FVC 2000 DB2a)
Fingerprint (one finger) [55]	10	FMR = 1.52, FNMR = 20.35 (FVC 2002 DB2)
This work (two fingers)	50	EER = 0.04 (FVC 2000 DB2a)
		EER = 0.22 (FVC 2002 DB1a)
